# Delivering biochemicals with precision using bioelectronic devices enhanced with feedback control

**DOI:** 10.1371/journal.pone.0298286

**Published:** 2024-05-14

**Authors:** Giovanny Marquez, Harika Dechiraju, Prabhat Baniya, Houpu Li, Maryam Tebyani, Pattawong Pansodtee, Mohammad Jafari, Alexie Barbee, Jonathan Orozco, Mircea Teodorescu, Marco Rolandi, Marcella Gomez

**Affiliations:** 1 Applied Mathematics, Baskin School of Engineering, University of California, Santa Cruz, CA, United States of America; 2 Electrical and Computer Engineering, Baskin School of Engineering, University of California, Santa Cruz, CA, United States of America; 3 Department of Earth and Space Sciences, Columbus State University, Columbus, GA, United States of America; Vellore Institute of Technology, INDIA

## Abstract

Precision medicine endeavors to personalize treatments, considering individual variations in patient responses based on factors like genetic mutations, age, and diet. Integrating this approach dynamically, bioelectronics equipped with real-time sensing and intelligent actuation present a promising avenue. Devices such as ion pumps hold potential for precise therapeutic drug delivery, a pivotal aspect of effective precision medicine. However, implementing bioelectronic devices in precision medicine encounters formidable challenges. Variability in device performance due to fabrication inconsistencies and operational limitations, including voltage saturation, presents significant hurdles. To address this, closed-loop control with adaptive capabilities and explicit handling of saturation becomes imperative. Our research introduces an enhanced sliding mode controller capable of managing saturation, adept at satisfactory control actions amidst model uncertainties. To evaluate the controller’s effectiveness, we conducted *in silico* experiments using an extended mathematical model of the proton pump. Subsequently, we compared the performance of our developed controller with classical Proportional Integral Derivative (PID) and machine learning (ML)–based controllers. Furthermore, *in vitro* experiments assessed the controller’s efficacy using various reference signals for controlled Fluoxetine delivery. These experiments showcased consistent performance across diverse input signals, maintaining the current value near the reference with a relative error of less than 7% in all trials. Our findings underscore the potential of the developed controller to address challenges in bioelectronic device implementation, offering reliable precision in drug delivery strategies within the realm of precision medicine.

## Introduction

Precision medicine customizes treatments by considering individual variations in patient responses. Strategies for treatment determination encompass factors like genetic mutations, age, and dietary influences [1]. An approach to precision medicine that further leverages advanced drug delivery techniques that integrate the concepts of location (specific tissues or organs) and timing (controlled release) has the potential to address existing drug-related challenges, thereby, enhancing the precision and effectiveness of therapies [2]. To this end, effective precision medicine requires medical devices capable of precise drug administration. This necessitates closed-loop control systems that can adapt treatment strategies based on real-time data about the biological system’s response and progression.

Recent developments in bioelectronics hold promise for advancing precision medicine [3–5] since they can serve as both sensors and actuators to enable capabilities for feedback control. Bioelectronic devices have been created that allow for monitoring blood sugar levels, controlling stem cell fate, applying electrical stimulation, and delivering therapeutic drugs [6–8]. Devices such as ion pumps in bioelectronics hold the potential for targeted drug delivery in therapy. Ion pumps are bioelectronic devices that allow for the movement of ions and charged drugs toward a targeted area electrophoretically [9]. Furthermore, one can enhance the capabilities of bioelectronic devices with machine learning and feedback control [10, 11]. Such capabilities can advance methods in precision medicine when applying a concentration of a drug at a specific region and time is more effective than passive drug delivery methods such as digested pills [12]. Such applications require the ability to carefully control the delivery of therapeutics with precision.

Implementing feedback control in bioelectronic devices presents unique challenges including variability in device performance and a limited operating range to name a few. In general, it is difficult to fabricate bioelectronics in a way that the end products are identical. Due to variations occurring in the fabrication process, the bioelectronic devices can differ in their properties. Components in the device can also vary in their performance when the device is used for an extended period of time [13]. This can lead to differing responses across devices and time when used in an experimental setting. To address the above-mentioned challenges, in previous work, a neural network (NN)-based machine learning algorithm was used with ion pumps to successfully control the pH level of a target solution [14]. A feedback control algorithm was able to regulate pH levels according to a target time-varying trajectory. In [15], the authors were able to use a similar control algorithm to control the membrane potential of stem cells by way of regulating the pH of the extracellular environment. Thus, an adaptive feedback control algorithm helps to mitigate variations in system response even when they evolve in time. While these algorithms show promise in regulating device behavior without predictive models, explicit handling of saturation issues remains necessary to improve controller performance. That is, the voltages that can be applied to the device are limited needs to be taken into account. In [16], the authors presented an alternative approach to arriving at an adaptive algorithm that has the benefits of the NN-based controller but explicitly handles saturation with guaranteed convergence. This algorithm was tested *in silico* using a mathematical model of an ion pump for controlled delivery of *H*+ ions to regulate the pH level of a target solution.

This paper aims to bridge the gap between bioelectronics, precision medicine, and drug delivery control. This study seeks to extend the application of control algorithms, adapting them for the precise delivery of Fluoxetine using ion pumps. As evidenced in prior research, Fluoxetine, primarily used in psychiatric disorders, presents intriguing immune-modulatory properties [17]. Notably, Fluoxetine exhibits the capacity to modulate immune responses in patients with depression, impacting lymphocyte activity linked to dysfunctional serotonergic systems [18]. Consequently, studies investigated the potential repurposing of topically applied Fluoxetine to enhance wound healing in diabetic mice [19] by targeting multiple facets of the healing process. These studies demonstrated Fluoxetine’s ability to promote re-epithelialization while concurrently mitigating inflammation, thereby offering a promising avenue for improving healing outcomes.

In this paper, we present an adaptation of the algorithm presented in [16] to control the amount of Fluoxetine biochemical being delivered by an ion pump. In our adaptation, we apply a heuristic switching algorithm for modification of the controller gains to improve performance and better adaptability for *in vitro* study. First, we demonstrated the applicability of the improved controller *in silico* by applying it to control an extended mathematical model of the proton pump developed in our previous work [16]. We compared the performance of the developed controller with the classical Proportional Integral Derivative (PID) controller as well as a machine learning (ML)–based controller to further validate its applicability. Later, we verified the applicability of the improved controller *in vitro* with three different reference signals that resulted in the delivery of the same total drug (Fluoxetine) concentration throughout the period of actuation. This was done to show the controller’s ability to keep the current value of the device at the reference regardless of the signal used as well as to allow for different methods of delivery. We demonstrate that the controller is able to perform at a reasonable level (a relative error of less than 7% in all the experiments), keeping the current near the reference throughout the experiment.

The paper will be structured as follows. Subsection “Bioelectronic Ion Pump” will introduce the ion pump and its capability to deliver Fluoxetine. Subsection “Controller” will discuss the sliding mode controller being used. Subsection “Experimental Set up” will discuss the experimental setup and how things are integrated together. Sections “Results” and “Discussion” will show the results and a discussion on them.

## Materials and methods

In this section, we introduce the ion pump and discuss its ability to deliver Fluoxetine. We then introduce an extended mathematical model of the bioelectronic ion pump for *in silico* study. Later, we discuss the sliding mode controller and give a brief outline of how the controller works. Finally, we present an overview of the experimental setup.

### Bioelectronic ion pump

Ion pumps are bioelectronic devices that can perform precise electrophoretic delivery of ions and molecules from a region of high concentration in the device, known as the reservoir, to the region of interest, known as the target [9, 20]. Over the years, ion pumps have been used to modulate pH in a local environment [21], treat neurological disorders [22, 23], induce macrophage recruitment [24] and even to deliver biochemicals in plants [25]. In this work, we use the ion pump to deliver Fluoxetine, a biochemical which is a selective serotonin reuptake inhibitor known to have immunomodulatory effects [19].

**Fig 1:**
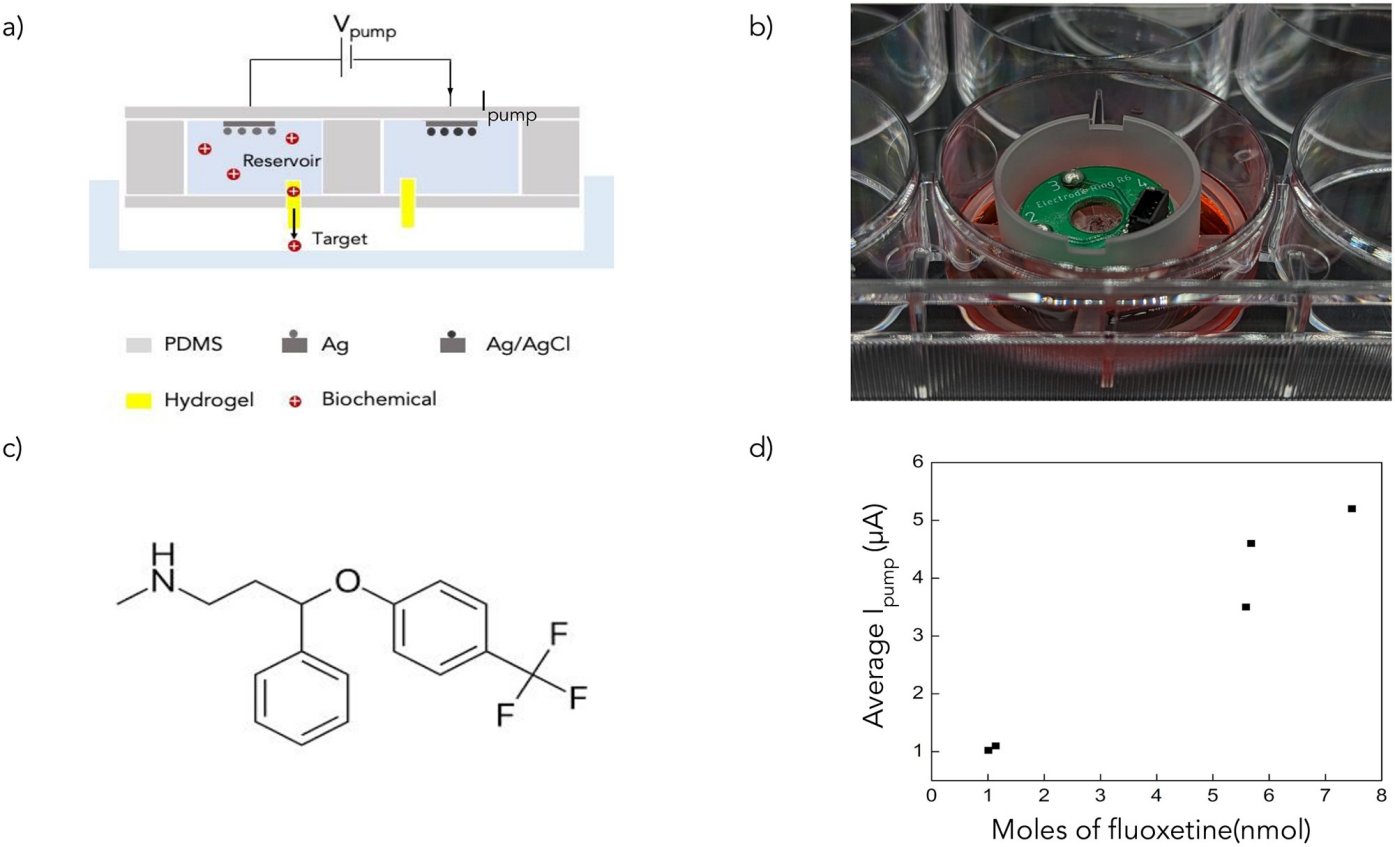
(a) Schematic of the bioelectronic ion pump. (b) Image of the ion pump in a 6-well plate with buffer solution. (c) Chemical structure of Fluoxetine (d) Graph showing the relationship between the I_pump_ and the amount of Fluoxetine delivered.

The ion pump transfers the biochemical from the reservoir to the target, with a voltage (V_pump_, typically between 0.5*V* and 2*V*) applied between the working electrode (Ag) and the reference electrode(Ag/AgCl) (see Fig 1(a)). A negatively charged hydrogel selectively transports the biochemical between the target and the reservoir. In the reservoir, we have 0.01*M* of Fluoxetine hydrochloride. Fluoxetine exists as a positively charged species at a pH of 5. For a positive V_pump_, the Fluoxetine moves through the hydrogel-filled capillary and is transported to the target [26]. Fig 1(b) shows an image of the device in a six-well plate with a buffer solution. This acts as our target for the experiments.

We collect the target solution after completion of delivery and use High-Performance Liquid Chromatography (HPLC) to estimate the amount of Fluoxetine in the target. We establish a relationship between the current produced during the biochemical delivery (I_pump_) and the corresponding amount of Fluoxetine delivered to calibrate the ion pump’s performance (see Fig 1(d)). Based on these results, we found the devices to be delivering the biochemical with an efficiency of approximately 20%, where efficiency is defined as follows

$$\begin{array}{c} \eta =\frac{\text{Number}\;\text{of}\;\text{moles}\;\text{of}\;\text{Fluoxetine}\;\text{delivered}}{\text{Number}\;\text{of}\;\text{moles}\;\text{of}\;\text{electrons}\;\text{transferred}}\times 100\% \end{array}$$ (1)

The number of moles of electrons transferred is obtained by integrating I_pump_ over the duration of delivery.

### Mathematical model of bioelectronic ion pump

To demonstrate the ability of the sliding mode controller to control the current value in an ion pump and to verify its stability, we present an extended mathematical model of the proton pump based on the one developed in [16] and use the same data for parameter fitting. The extended mathematical model is as follows

$$\begin{array}{c} \begin{array}{cc} u>\epsilon , & \left\{ \begin{array}{c} {\dot{x}}_{1}=D\left(x_{2}-x_{1}\right)+u\frac{c_{1}x_{2}}{d_{1}+x_{2}}-gx_{1}\\ {\dot{x}}_{2}=D\left(x_{1}-x_{2}\right)+u\frac{c_{1}x_{2}}{d_{1}+x_{2}} \end{array}\right.\\ \epsilon \geq u>0, & \left\{ \begin{array}{c} {\dot{x}}_{1}=D\left(x_{2}-x_{1}\right)+u\frac{cc_{1}x_{2}}{dd_{1}+x_{2}}-gx_{1}\\ {\dot{x}}_{2}=D\left(x_{1}-x_{2}\right)+u\frac{cc_{1}x_{2}}{dd_{1}+x_{2}} \end{array}\right.\\ 0\geq u\geq -\zeta , & \left\{ \begin{array}{c} {\dot{x}}_{1}=D\left(x_{2}-x_{1}\right)+u\frac{cc_{2}x_{1}}{dd_{2}+x_{1}}-gx_{1}\\ {\dot{x}}_{2}=D\left(x_{1}-x_{2}\right)+u\frac{cc_{2}x_{1}}{dd_{2}+x_{1}} \end{array}\right.\\ -\zeta >u, & \left\{ \begin{array}{c} {\dot{x}}_{1}=D\left(x_{2}-x_{1}\right)+u\frac{c_{2}x_{1}}{d_{1}+x_{1}}-gx_{1}\\ {\dot{x}}_{2}=D\left(x_{1}-x_{2}\right)+u\frac{c_{2}x_{1}}{d_{2}+x_{1}}. \end{array}\right. \end{array} \end{array}$$ (2)

where *x*_1_ is the ion concentration in the area where we are trying to control the mean fluorescence intensity and *x*_2_ is the ion concentration in the ion reservoir and ion bridge. Variable *D* is the natural diffusion rate of the dye into the media. The variable *u* is the applied voltage to the device. The parameters *c*_*i*_ and *cc*_*i*_ are the maximum rates of change of the ions upon actuation. Parameters *d*_*i*_ and *dd*_*i*_ are the half maximal values of the hill function capturing bounds on ion transfer rates. Although *x*_1_ is the ion concentration in only a small portion of where the image is taken, diffusion should still be accounted for since ions can still flow freely through the area. The variable *g* is added as a leakage term. To separate the model into four regions based on the range of voltages applied to the device, the variables *ϵ* and *ζ* are introduced.

### Controller

Sliding mode control is known for its ability to handle system uncertainties including unmodelled dynamics [27, 28]. A sliding mode controller attempts to achieve a reference signal by forcing the system to slide toward a stable manifold. The controller drives the system towards a designed sliding manifold, where the system stays, sliding along the manifold until it reaches a stable equilibrium. One major drawback with the control design is the chattering of the control signal that tends to appear [29, 30]. However, researchers have developed many methods to address this so the control method can be used in real-world applications. It is applied in robotics, process control, induction motors, and power converters [31–34].

**Fig 2:**
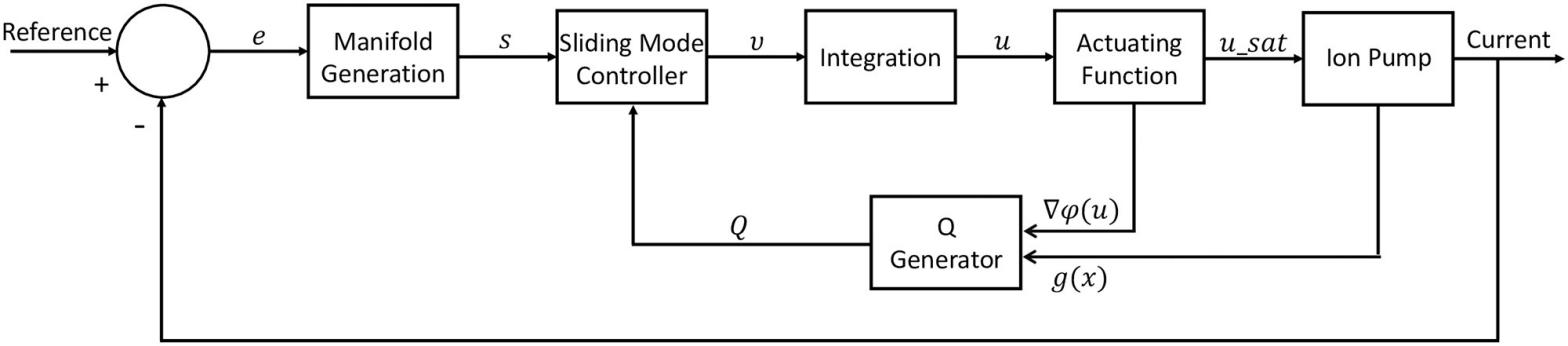
Closed-loop control architecture for automated ion pump actuation to achieve prescribed current values and regulate the delivered concentration of Fluoxetine.

A sliding mode controller similar to the one developed in [16] is utilized for the output feedback control experiment in this paper (see Fig 2). The controller developed in [16] was shown to perform well with a mechanistic model of an ion pump that included saturation. The challenges encountered in simulations are comparable to the ones expected in these experiments. This inspired confidence that the controller developed in [16] would be a viable controller.

To begin, we consider our system to be an affine nonlinear system with input saturation represented by the following state space model

$$\begin{array}{c} \left\{ \begin{array}{c} \dot{x}=f\left(x\right)+g\left(x\right)\phi \left(u\right)+\delta \left(t\right)\\ \dot{u}=\nu \\ y=h(x), \end{array}\right. \end{array}$$ (3)

where $x\in R^{n}$ is the state vector. In our experiment, *x* is the current readout from the device. An artificial control signal $\nu \in R^{m}$ is introduced to help reduce the known chattering issue with sliding mode controllers. The output of the function $\text{sat}\left(\phi \left(u\right)\right)\in R^{m}$ is the control input vector. In this application, this is the voltage applied to the device. The variable $y\in R^{n}$ is the output vector of the system. In the experiment, *y* = *x* is the measured current from the device. The function $f\left(x\right)\in R^{n}$ is an unknown locally Lipschitz nonlinear function and $g\left(x\right)\in R^{n\times m}$ is an unknown input coefficient value. The time-varying variable δ(t)∈R is a sufficiently smooth disturbance occurring throughout the experiment. We design the manifold as

$$\begin{array}{c} s=Ke+\dot{e}, \end{array}$$ (4)

where *K* is a positive constant, and *e* is the error defined as

$$\begin{array}{c} e(t)=x(t)-r(t), \end{array}$$ (5)

*x*(*t*) is the current value, and *r*(*t*) is the desired reference value for the current.

The sliding mode controller then calculates the artificial control input to be used for calculating the actual control signal to be sent to the ion pump

$$\begin{array}{c} \nu =\mu \rho \;\text{sign}(Qs), \end{array}$$ (6)

where *ρ* is a positive coefficient and *Q* is obtained as

$$\begin{array}{c} Q=g\left(x\right)\nabla_{u}\phi \left(u\right). \end{array}$$ (7)

We require the sign of *g*(*x*) found through experiments to be *μ* = −1, which is the case when the response of the system is monotonically increasing with the input. In some cases, the rate of change in the response could be faster or slower in one direction than the other. Therefore, we introduce a heuristic switching Algorithm 1 for better adaptation to these conditions. The actuating function used here is defined as

$$\begin{array}{c} \phi \left(u\right)=A_{max}\text{sin}\left(u\right), \end{array}$$ (8)

where *A*_*max*_ is the Min/Max voltage allowed by the ion pump. For the experiments, we set *A*_*max*_ = 3. To help reduce the chattering signal that tends to occur with sliding mode controllers, *ν* is integrated to get *u*. Integration is done using the trapezoidal rule where we have a sampling time of *T* = 1. It should be noted that using the trapezoidal rule requires us to start the algorithm at *t* = 2. Finally, to make sure we stay in the saturated region, we applied *ϕ*(*u*) to get the final voltage that will be applied to the ion pump

$$\begin{array}{c} u_{sat}=\phi \left(u\left(t\right)\right). \end{array}$$ (9)

**Algorithm 1** Gain Update Algorithm

Set parameters *κ*_1_, *κ*_2_, *ρ*_1_, and *ρ*_2_

**if** *e*(*i*) > 0 **then**

 Set *K* = *κ*_1_ and *ρ* = *ρ*_1_

**else**

 Set *K* = *κ*_2_ and *ρ* = *ρ*_2_

**end if**

Algorithm 2 shows a detailed description of the implementation of the developed algorithm. For details regarding the stability proof of the developed controller, readers are referred to our previous work [16].

**Algorithm 2** Sliding Mode Control Algorithm

Set parameters *n*, *r*(*i*), *T*, *μ*, *u*(1)

**for** *i* = 2: *n*, **do**

 Read the current output *y*(*i*)

 *e*(*i*) = *y*(*i*) − *r*(*i*)

 Call **Algorithm** 1

 $s\left(i\right)=Ke\left(i\right)+\dot{e}$

 *ν*(*i*) = *μρ* sign(*s*(*i*)*A*_*max*_ cos(*u*_*i*−1_))

 $u\left(i\right)=u\left(i-1\right)+(\frac{\nu (i)+\nu (i-1)}{2T})$

 *u*_*sat*_(*i*) = *A*_*max*_ sin(*u*(*i*))

**end for**

### Experimental set up

The ion pump is integrated with a printed circuit board (PCB) interface which connects the electrodes in the pump to an external power supply. We use silver pins and a conductive silver paste to connect the Ag/AgCl electrodes in the reservoir to the PCB which is then soldered to the ion pump. Jumper cables are used to connect this PCB to a Raspberry Pi-based external voltage controller which can connect to the WiFi [35]. The device is seated in a six-well plate with a buffer solution acting as the target for delivery [24]. The external voltage controller applies the control commands it receives from the sliding mode controller to the ion pump. The setup is able to successfully provide up to 16 different V_pump_ values independently through 16 channels, with a resolution of 1.95*mV* per channel. It also simultaneously records I_pump_ values from each channel, with a resolution of 0.125*nA*. This resolution could lead to extra uncertainty in the system.

**Fig 3:**
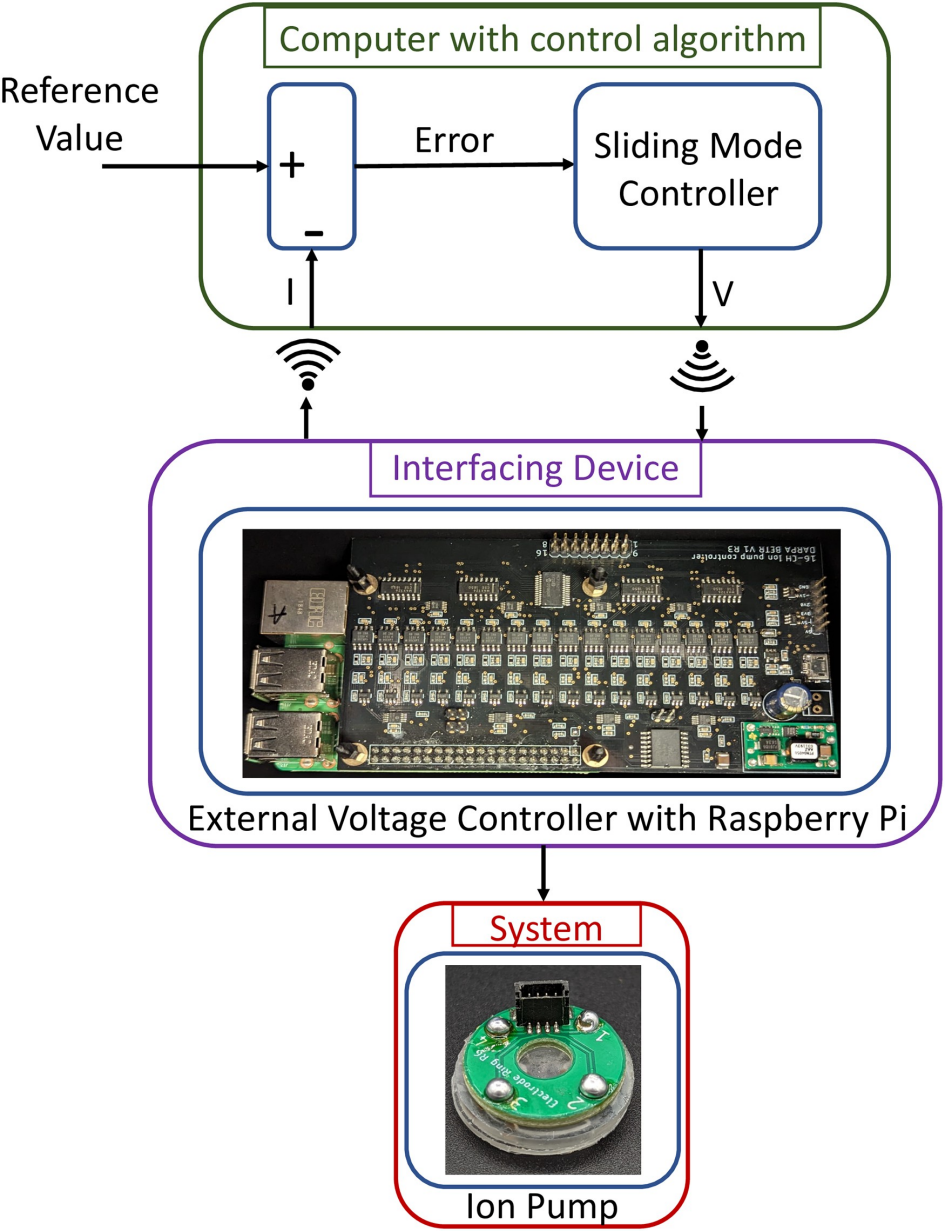
Schematic of the experimental closed-loop setup. The error is computed using the values of the current output read from the ion pump and the desired reference value. The sliding mode controller evaluates the next voltage value. This is sent to the external voltage controller with Raspberry Pi through a WiFi connection with the laptop running the control algorithm. The external voltage controller applies the value through a connected cable from the ion pump. After the voltage has been applied, the external voltage controller reads the current from the ion pump and sends it back to the laptop with the control algorithm through WiFi closing the loop.

Fig 3 shows the closed-loop experimental setup. The error is calculated using the value of the current output read from the ion pump and the desired reference value. The sliding mode controller evaluates the next voltage value to drive the current toward the desired reference. This is sent to the Raspberry Pi through a WiFi connection between the Raspberry Pi and a laptop where the control algorithm is running. The Raspberry Pi applies the voltage value to the ion pump through a connected cable. This closes the loop.

## Results

We performed several experiments to validate the performance of the developed controller. Table 1 shows the control parameters used in the *in silico* and *in vitro* experiments. In this study, the developed control algorithm was implemented utilizing a MacBook Pro equipped with a 2.3 GHz Quad-Core Intel Core i7 processor, 32 GB of 3733 MHz LPDDR4X memory, running on the Big Sur operating system.

**Table 1:** Detailed control design parameters of the SMC used in *in silico* and *in vitro* experiments.

	*in silico*	*in vitro*
Sampling Time (*T*)	1[*s*]	1[*s*]
*μ*	−1	−1
*κ*_1_, *κ*_2_	0.1, 0.05	0.4, 0.4
*ρ*_1_, *ρ*_2_	0.01, 0.07	0.08, 0.008
*u*(1)	0	0
Total Experiment Time (*n*)	1200[*s*]	1200[*s*]
*A*_*max*_	1.5	3

### In silico

The performance of the sliding mode controller is shown using the mathematical model of the ion pump device. It is reasonable to assume that a large enough value for the design parameter *ρ* will allow for stability. The drawback of using a large value for *ρ* will be an increased amount of chattering in the control signal, but it is an acceptable trade off for stability. The ion pump response to step changes in the reference signal starting at 17.5 dropping by 0.5 each 400 seconds is considered as control objective for the *in silico* study. We compared the performance of the developed controller with the classical Proportional Integral Derivative (PID) controller as well as a machine learning (ML)-based controller to further validate the benefits of explicitly considering saturation.

**Fig 4:**
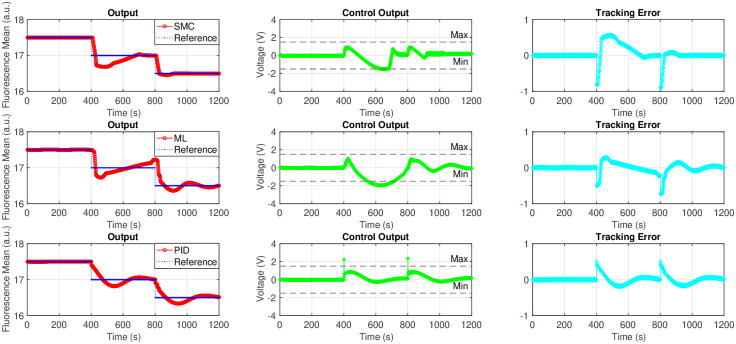
*In silico* results for feedback control on fluorescence mean pixel value in the mathematical model of ion pump device using sliding mode control (SMC), machine learning (ML)–based control, and PID control. The first row shows the ion pump response to step changes in the reference signal starting at 17.5 dropping by 0.5 each 400 seconds using SMC. The middle row shows the ion pump response to step changes in the reference signal starting at 17.5 dropping by 0.5 each 400 seconds using ML-based control. The last row shows the ion pump response to step changes in the reference signal starting at 17.5 dropping by 0.5 each 400 seconds using PID. The blue line in the first column indicates the desired fluorescence mean pixel value set as a reference for the feedback control algorithm. The red curve represents the output of the mathematical model (fluorescence mean pixel value). The second column shows the control output that is applied to the model in green. The third column shows the error between the desired and measured fluorescence mean pixel value referred to as tracking error in cyan.

The results of the *in silico* study are shown in Fig 4. The left column plots are the system responses in blue and the reference being tracked in red. The middle column plots show the control effort generated by each controller. The right column plots show the tracking error. The tunable parameters in all controllers were chosen such that we can evaluated the performance of the controller near saturation values. It is clearly seen that the SMC does not violate the Min/Max value in its generated control output while the other two controllers generated control values outside the Min/Max value range. This is because no saturation handling mechanisms were incorporated into the other two control strategies. It is worth noting that, we applied a “hard limit” to manually clip the generated signal at Min/Max value threshold for the PID and ML–based controllers. This is a necessary safety action. The sliding mode controller does chatter slightly but keeps the system the fluorescence intensity near the desired trajectory. We see that the error is kept low throughout all simulations.

### In vitro

**Fig 5:**
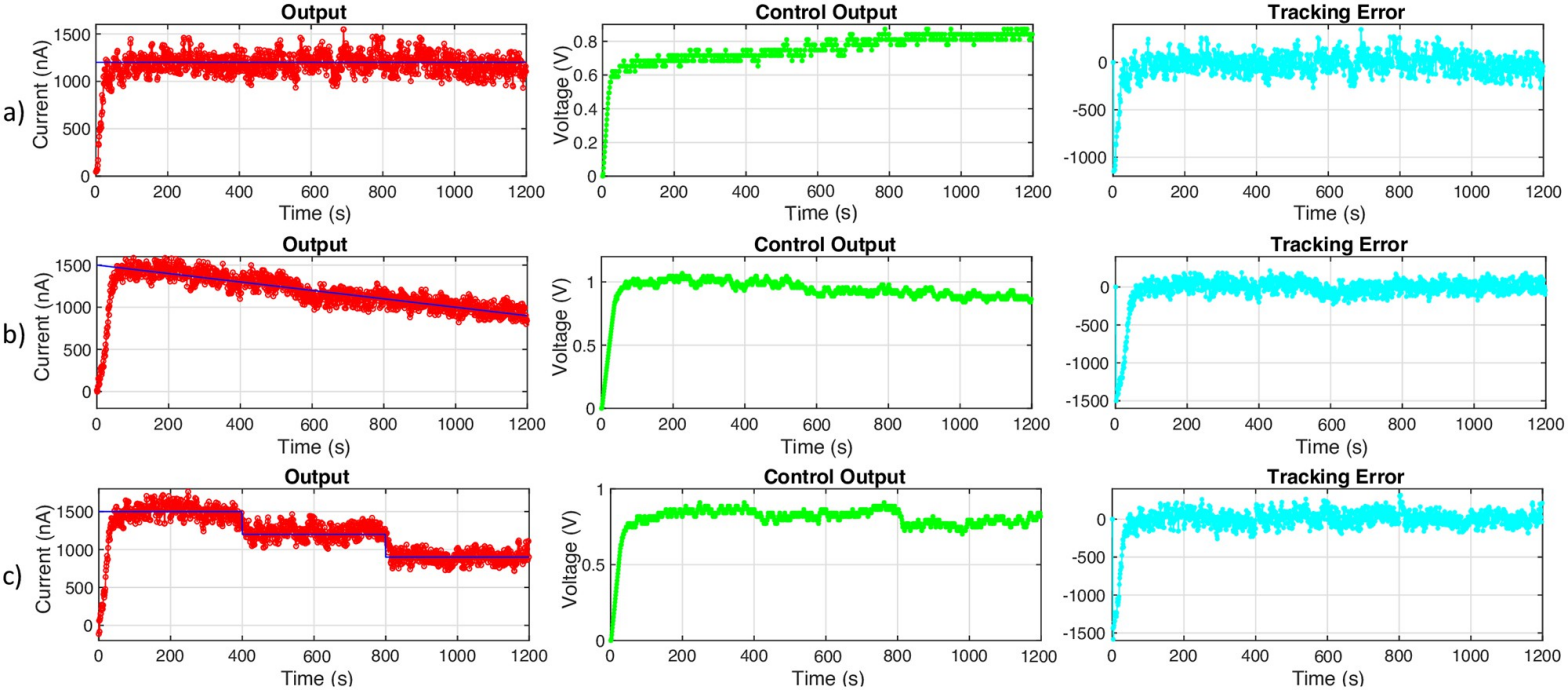
Experimental results for feedback control on current in the ion pump device using sliding mode control. Row (a) shows the ion pump response to a constant reference signal at 1200*nA*. Row (c) shows the ion pump response to step changes in the reference signal starting at 1500*nA* dropping by 300*nA* each 400 seconds. Row (b) shows the ion pump response to a gradual decline reference signal beginning at 1500*nA* and ending at 900*nA*. The blue line in the first column indicates the desired current set as a reference for the feedback control algorithm. The red curve represents the measured current from the device in real-time. The second column shows the control output that is delivered to the device in green. The third column shows the error between the desired and measured current referred to as tracking error in cyan.

Fig 5 shows experimental results for feedback control on current in the ion pump device using sliding mode control. In the first column, the blue lines indicate the desired current set as a reference for the feedback control algorithm. The red line is the current being measured from the device in real-time. The second column shows the controller output (i.e., voltage) being applied to the ion pump in green. The third column shows the corresponding tracking error in cyan.

We establish a value for the reference signal by estimating the amount of current required to deliver a dosage of 0.45*mg* Fluoxetine. Based on device calibration, we estimate that a current of 1.2*μA* (1200*nA*) is required to meet the desired dosage of biochemical.

In the first experimental setup (see Fig 5(a)), we test the controller’s performance on a constant reference signal of 1200*nA*. In the second experiment (see Fig 5(b)), we test the controller’s performance on a gradual descending reference signal starting at 1500*nA* dropping to 900*nA* by the end of the experiment. In the third experiment (see Fig 5(c)), we test the controller’s performance on a step-wise descending reference signal starting at 1500*nA*, dropping by 300*nA* every 400 seconds. Note that the fluctuations around the reference signals at steady-state have a relative error of less than 7% in all the experiments. We define the system to be at steady-state after the first 100 seconds of the experiment to allow for all three runs to reach the reference from the starting current of 0*nA*. Since the amount of biochemical delivered is determined by integrating over the measured current, we don’t expect that the high-frequency fluctuations in current measurements have a high impact on the overall uncertainty in the biochemical delivery. Although the fluctuations make it difficult to keep the current exactly at the reference, the average current throughout each experiment can be calculated to show that the desired amount of Fluoxetine is being delivered. The averages for each reference signal are as follows: constant—1171.49*nA*, gradual decline—1166.45*nA*, decreasing steps—1184.75*nA*. Table 2 shows the relative error information along with the averages.

**Table 2:** Quantitative measures of all the experiments.

Experiment	average output signal	average steady-state relative error
constant	1171.49*nA*	7%
gradual decline	1166.45*nA*	6%
decreasing steps	1184.75*nA*	7%

## Discussion

We see that the controller had to gradually increase the voltage applied to the ion pump in order for it to maintain the constant reference. This is due to the device’s decaying performance as the experiment continues. This highlights the need for an adaptive control strategy and reinforces why open-loop control is unable to deliver a desired concentration with precision. One thing to notice is that the average current throughout the experiment was always lower than the targeted average signal of 1200nA. This is likely due to the initial transients at the beginning of the experiment and should be considered when setting reference current values and duration.

An enhancement that can be applied to achieve better control of the ion pump is to develop an algorithm that automatically tunes the control parameters. The reason for this is that the efficiency of the ion pump tends to decline over time. This would mean that the response of the device will change over the course of the experiment, which could affect the performance of the sliding mode controller. Adaptive parameters could lead to better performance since they would adjust as needed to the varying response of the ion pump.

Some of the oscillatory behavior of the current could be due to the interfacing board used. When the controller sends a voltage value that is smaller than the resolution of 1.95*mV* then the board is unable to send the exact voltage value. This can cause slight oscillatory behavior with the current as small changes in voltage are able to cause large changes in the current as seen in Fig 5. Although exact control of the current at the reference is not achievable, we see in Table 2 that the average of the current is near the desired value and is within acceptable relative error bounds.

Finally, the presented work combining closed-loop control and delivery of Fluoxetine by ion pumps can have real world applications in the wound-healing field. People can respond differently to the same applied medical treatment for reasons such as age, ethnicity, and genetics [36]. This makes it difficult for physicians to treat people optimally. Through precision medicine, custom treatment options can be created depending on an individual’s needs. Closed-loop control allows for the customization of the treatment strategy by changing the strategy in real-time as new information is gained [37]. This can be done by interfacing the feedback control regulated ion pump presented here with a higher-level control algorithm that provides the reference signal [16]. In this way, wound healing might be treated by sensing how well a wound is responding to a specific treatment and changing the dosage of biochemicals as needed in real-time.

## Conclusion

In this study, we have addressed precise drug delivery using bioelectronic devices. Leveraging ion pumps for controlled Fluoxetine delivery has demonstrated a viable approach toward personalized therapeutics. The application of a sliding mode controller, specifically designed to manage the inherent limitations and uncertainties associated with bioelectronic devices, is demonstrated. Both *in silico* and *in vitro* experiments exemplify the robustness and efficacy of our enhanced sliding mode controller. Through *in silico* simulations using an extended mathematical model of the proton pump, we validated the controller’s capability to handle uncertainties while maintaining precise control over drug delivery. Subsequently, the *in vitro* experiment reinforced these findings, showcasing consistent drug delivery across various reference signals. The utilization of the ion pump’s current value as an indicator of drug dosage offers a tangible method for quantifying biochemical delivery. Moreover, our developed sliding mode controller, equipped with adaptive capabilities to handle saturation and model uncertainties, presents a promising solution to the challenges encountered in bioelectronic device implementation. The developed controller’s performance is compared to classical Proportional Integral Derivative (PID) and machine learning-based controllers. The potential for precise, adaptable, and reliable drug delivery strategies not only impacts precision medicine but also extends to fields like wearable or implantable bioelectronics.

## Supporting information

S1 File Step reference experimental data. This dataset corresponds to the experiment with declining steps in the reference trajectory being tracked. The experiment ran with a 1 second sampling time. The variables in the file are as follows: *d*- the desired trajectory (reference); *err*- the error from the current output and reference; *u*- the applied voltage calculated by the sliding mode controller; *plot*_*t*_*imer*- the time steps (in seconds).
(XLSX)

S2 File Declining reference experimental data. This dataset corresponds to the experiment with a decreasing linear reference trajectory being tracked. The experiment ran with a 1 second sampling time. The variables in the file are as follows: *d*- the desired trajectory (reference); *err*- the error from the current output and reference; *u*- the applied voltage calculated by the sliding mode controller; *plot*_*t*_*imer*- the time steps (in seconds).
(XLSX)

S3 File Constant reference experimental data. This dataset corresponds to the experiment with a constant reference trajectory being tracked. The experiment ran with a 1 second sampling time. The variables in the file are as follows: *d*- the desired trajectory (reference); *err*- the error from the current output and reference; *u*- the applied voltage calculated by the sliding mode controller; *plot*_*t*_*imer*- the time steps (in seconds).
(XLSX)
